# Fluoroscopy-Guided Metallic Foreign Body Removal: A Report of Three Cases and Literature Review

**DOI:** 10.7759/cureus.40462

**Published:** 2023-06-15

**Authors:** Thamer M AlBilasi, Lama F AlDhawi, Ahmed N AlOlaywi, Alyaa S Al Mutairy, Fareed R AlGhamdi, Saleh S Alamry, Hatem A AlZhrani

**Affiliations:** 1 Department of Otolaryngology - Head and Neck Surgery, Prince Sultan Military Medical City, Riyadh, SAU; 2 Collage of Medicine, AlMaarefa University, Riyadh, SAU; 3 Otolaryngology - Head and Neck Surgery, Security Forces Hospital, Riyadh, SAU

**Keywords:** air gun shot, fluoroscopy, head and neck, penetrating trauma neck, metallic foreign body

## Abstract

Ingested foreign objects that become trapped in the upper aerodigestive tract is a common issue that arises in Otolaryngology-Head and Neck Surgery practice. In these circumstances, it is advised to explore the neck using an external method to remove the item. However, locating the foreign body might be challenging. Not all metallic foreign body (MFB) patients require surgery, especially those without symptoms or complications. The standard X-ray and CT images are routinely examined for preoperative assessment and localization. Removal can be accomplished via flexible pharyngo-laryngoscopy or upper gastrointestinal endoscopy. Fluoroscopy is a widely accessible, minimally invasive, but underutilized tool during procedures. It offers an accurate intraoperative assessment of the foreign body in real-time. To allow the planning of a secure extraction pathway, the target should be radiopaque. In this report, we present three unique cases in which we used fluoroscopic imaging for guidance to remove a foreign body in the head and neck region in Prince Sultan Military Medical City in Riyadh, Saudi Arabia. In the first case, a young male presented with a history of foreign body sensation and odynophagia in the throat after eating a (shawarma) sandwich. In the second case, a six-year-old boy presented to the emergency department (ED) with epistaxis after being exposed to an air gun shot to his face. In the third case, a 40- year-old male presented after exposure to an air gun shot to the neck. After identification of the foreign body, all three patients were referred to Otolaryngology-Head and Neck. After radiological images have been done to confirm the presence of foreign objects, all three had a minimally invasive procedure to remove the metallic foreign bodies under fluoroscopic guidance without needing extensive surgery. All the procedures went well with no immediate complications with discharge on the same day. Fluoroscopy-guided removal of foreign bodies related to metabolic forging is a promising technique with several advantages, including real-time visualization, reduced invasiveness, and shorter recovery times. However, it is essential to weigh the benefits against the risks associated with radiation exposure and inherent limitations in detecting non-metallic objects. Further research and clinical studies are needed to optimize this technique and establish evidence-based guidelines for its application in the field of metabolic forging bodies.

## Introduction

An issue that frequently arises in Otolaryngology-Head and Neck Surgery practice is ingested foreign objects that become trapped in the upper aerodigestive tract. Discovering the item is the major challenge. Extraluminal migration of ingested foreign objects is uncommon. In these circumstances, it is advised to explore the neck using an external method to remove the item. However, locating the foreign body might be challenging [1]. Despite being seen as a toy, air guns can result in minor to severe injuries. The damage could seem minor, but it could occasionally result in serious morbidity or death. The kind of air pistol used, the distance at which it is fired, the projectile's velocity, and the anatomic location of penetration where the pellet impacts affect the kind and seriousness of injuries [2]. Metallic objects in the human body are typically brought on by iatrogenic occurrences, industrial accidents, war, and other catastrophes. Not all metallic foreign body (MFB) patients require surgery, especially those without symptoms or complications [3]. 

In a foreign body occurrence, the standard radiologic and computed tomography (CT) images are routinely examined, and they can provide some insight into the whereabouts of the missing item. In order to find and remove foreign objects in the head and neck region, video endoscopy has also been developed. The introduction of computer-assisted surgery (CAS) for the three-dimensional localization of foreign bodies has proved invaluable [4]. Removing foreign bodies from the head and neck region may be significantly aided by using a computer-based image-guided system. It reduces surgical time, permits minimally invasive access, and helps to avoid serious complications including damage to nearby important structures [5]. 

Metal, gravel, and glass can all be successfully found using mini-C-arm fluoroscopy, but wood or plastic cannot [6]. Injury to the head and neck from non-powder firearms carries a risk of significant mortality and morbidity. Particularly in children, such penetrating injuries frequently go unreported or are minimized. Damage from air gun pellets can affect both soft tissue and bone. Some metals may cause a foreign body reaction or produce toxins over time if they become embedded in body tissue. Recovering these pellets is therefore essential [7]. Approximately 70% of cases of foreign bodies in the paranasal sinuses are related to maxillofacial trauma, while other cases are connected to maxillary dental treatments. Only 2% of individuals with penetrating head and neck trauma had foreign bodies left in their paranasal sinuses or skull bases, according to research. Air gun-related sinus injuries make up a minor portion of head and neck penetrating wounds [8]. Air gun-related ballistic injuries with retained foreign bodies are a rather prevalent issue, especially in kids and teenagers. Foreign bodies can cause complications, including pain and infection if they are not promptly removed. The entire spectrum of radiology's diagnostic techniques, in particular radiography, ultrasonography (USG), and computed tomography (CT), are used to detect the presence of foreign bodies. Primary care and emergency doctors, surgeons, and radiologists can remove foreign entities with or without imaging guidance. Assessing and evaluating the methods and modalities of image-guided detection and removal of ballistic foreign bodies secondary to air gun hits, which is a very effective procedure within the interventional radiology department, are important and can obviate the need for open surgical procedures [9]. 

The head and neck regions are complex-shaped anatomical areas that contain numerous delicate organ systems, including the neurological, vascular, and aerodigestive systems, as well as the auditory and visual systems. The head, neck, and face, in particular, are frequently exposed to the outside world, much like the hands. Therefore, compared to other (often clothed) body parts, injuries to these areas are more frequent and pose a larger risk of foreign body contamination. The materials that are used the most frequently are glass, metal, and wood. Foreign bodies can enter the head and neck through the mouth, nostrils, or external auditory meatus in addition to penetrating the skin or the eyeball. They might extend into the orbits, paranasal sinuses, or the deep head and neck areas [10]. 

There have been numerous reports of events involving foreign bodies that were either unintentional, self-inflicted, iatrogenic, or assault-related. Inhalation, swallowing, and direct penetration are various trauma causes. Acute and sometimes fatal complications from foreign body injuries might include bleeding, compromised airways, and neurovascular damage. There is a general danger of late complications, primarily infections, which can lead to permanent damage, even if some residual foreign bodies may stay clinically silent for years or even forever. Delayed problems can include pain that doesn't go away, poor wound healing, and inflammatory reactions that can lead to fistulas, necrotizing fasciitis, abscesses, and the migration of foreign objects [10]. 

## Case presentation

Case 1

An otherwise healthy six-year-old boy presented with a history of an air-gun pellet shot on the face. The patient complained of nose bleeding and pain with one episode of vomiting. There was no loss of consciousness, no abnormal movement, and no visual changes. On clinical examination, it showed left eye ecchymosis and edema, mainly in the lower eyelid, with mild conjunctival redness, intact vision, and extraocular movement (EOM) movement. There was a small superficial entrance wound on the nasal dorsum with no tenderness or crepitation on the skull or spine or nose.

**Figure 1:**
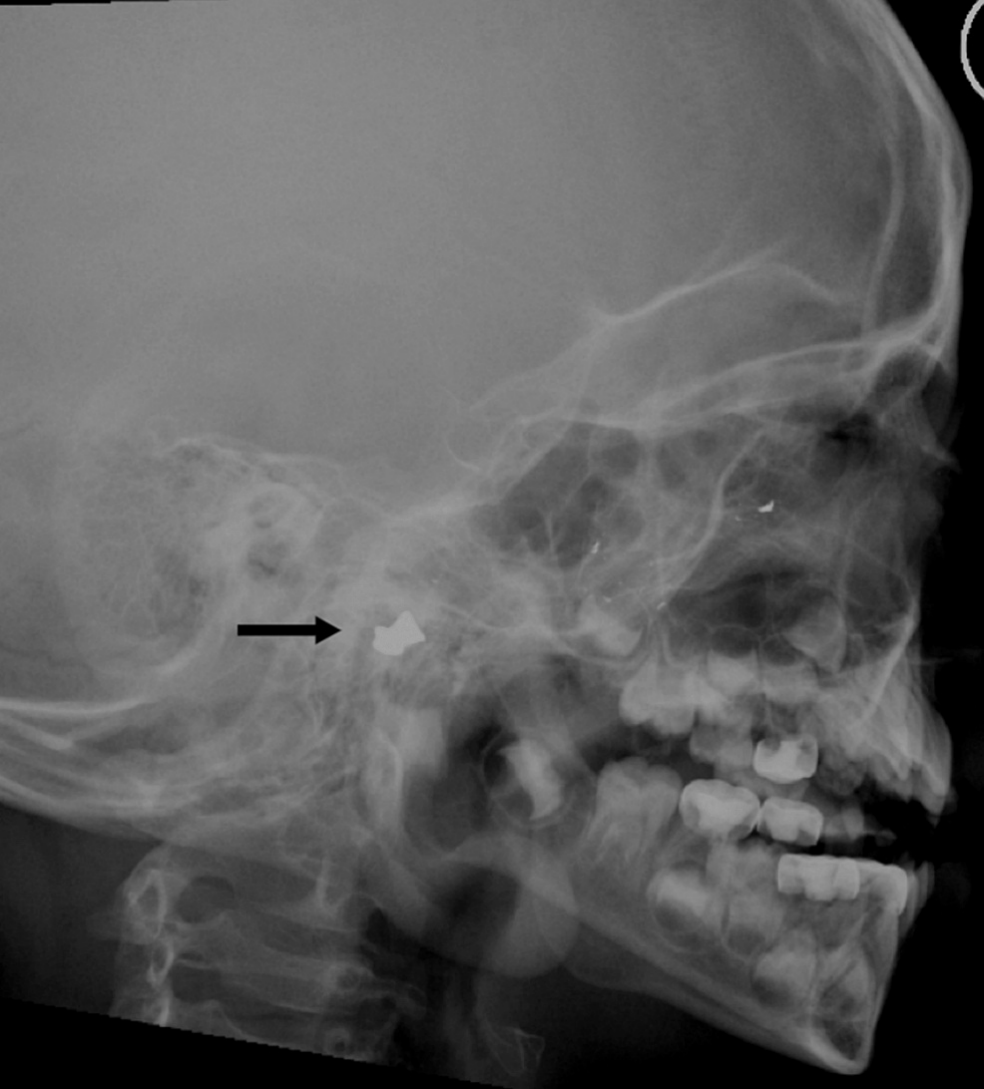
The black arrow points to the metallic foreign body

ENT and head and neck examinations were unremarkable. Head X-ray showed foreign body near the left temporomandibular joint, left maxillary sinus, and anterior ethmoid sinus. Multiple subcutaneous foreign bodies were noted (Figure 1).

The patient was taken to the operating room and, after general anesthesia induction, C-arm fluoroscopy guidance was requested and direct location to the foreign body was done using a spinal needle. Then, a small mucosal incision was done through the oral cavity measuring less than 0.5 cm, with the uneventful removal of the MFB under fluoroscopy guidance. The sinus part of the MFB was removed with nasal endoscopy. On postoperative day 1, the patient was seen as doing well and discharged home in good condition.

Case 2

A 20-year-old male presented with foreign body sensations that started three hours prior. The patient reported this feeling started after eating a shawarma sandwich after which he immediately presented to the emergency department.

**Figure 2:**
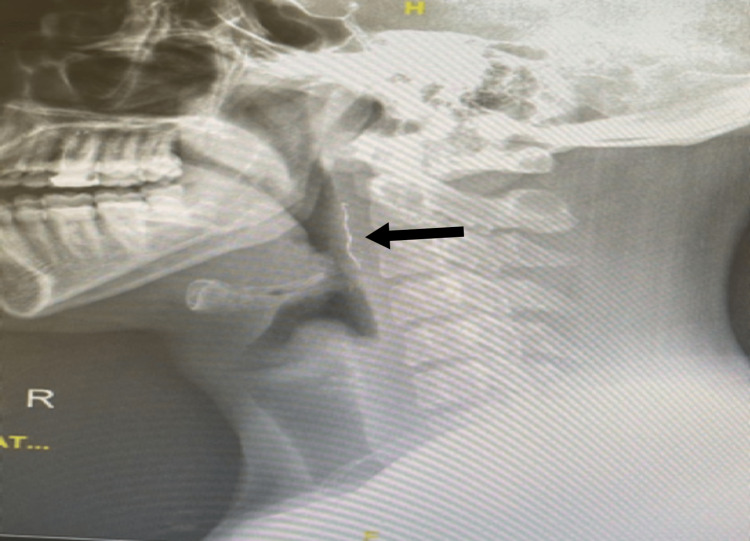
The arrow points to the metallic foreign body in the posterior pharyngeal wall soft tissue

On clinical examination, there was no drooling, erythema, or edema of the oropharynx, posterior pharyngeal wall, nasopharynx, and hypo-pharynx. Flexible nasal endoscopy showed no abnormalities or foreign body. X-ray of the lateral neck shows a liner radiopaque shadow in the pre-vertebral soft tissue at the level of the cervical 1 - cervical 3 vertebral body (Figure 2)

**Figure 3:**
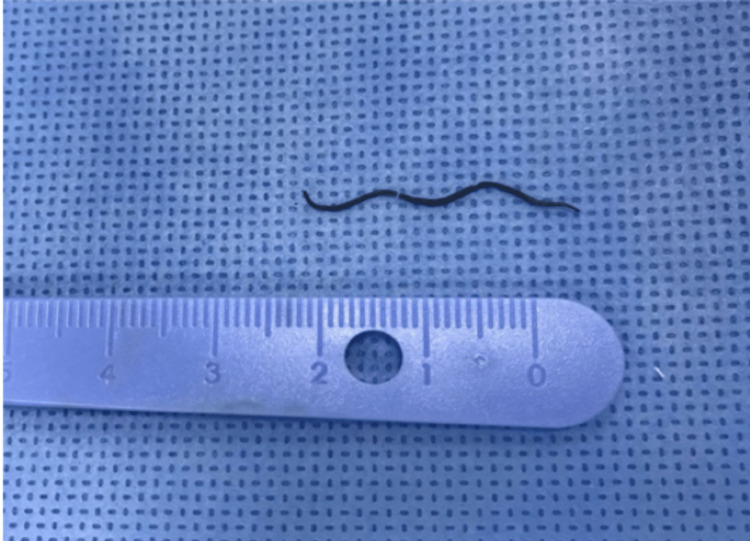
Metallic foreign body after removal

The laboratory tests were unremarkable. Consent for transoral foreign body removal vs transcervical approach was obtained. The patient was kept on nothing by mouth (NPO) and on intravenous fluid. In the operating room, after general anesthesia induction, under C-arm fluoroscopy guidance, there was an uneventful removal of the MFB (Figure 3) through a small mucosal incision measuring 0.5 cm in the posterior pharyngeal wall.

On postoperative day 1, the patient was seen doing well, tolerating oral feeding, and discharged home in good condition.

Case 3

**Figure 4:**
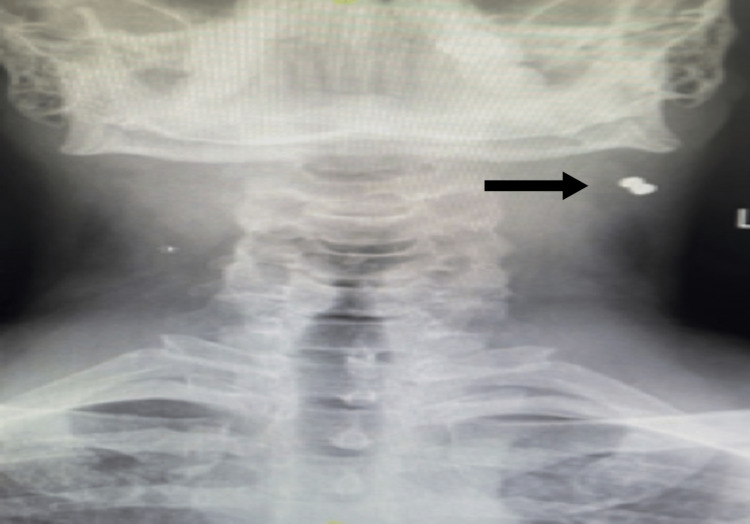
The arrow points to the metallic foreign body within the left sternocleidomastoid muscle

A 25-year-old healthy male presented with a history of air gun shot to the neck on level lIA. Clinical examination showed that there was an entrance wound on the left side of the neck with no exit wound, no bleeding, hematoma, or emphysema. ENT and head and neck examinations were unremarkable. A neck X-ray was done that showed a MFB on the left side of the neck (Figure 4).

**Figure 5:**
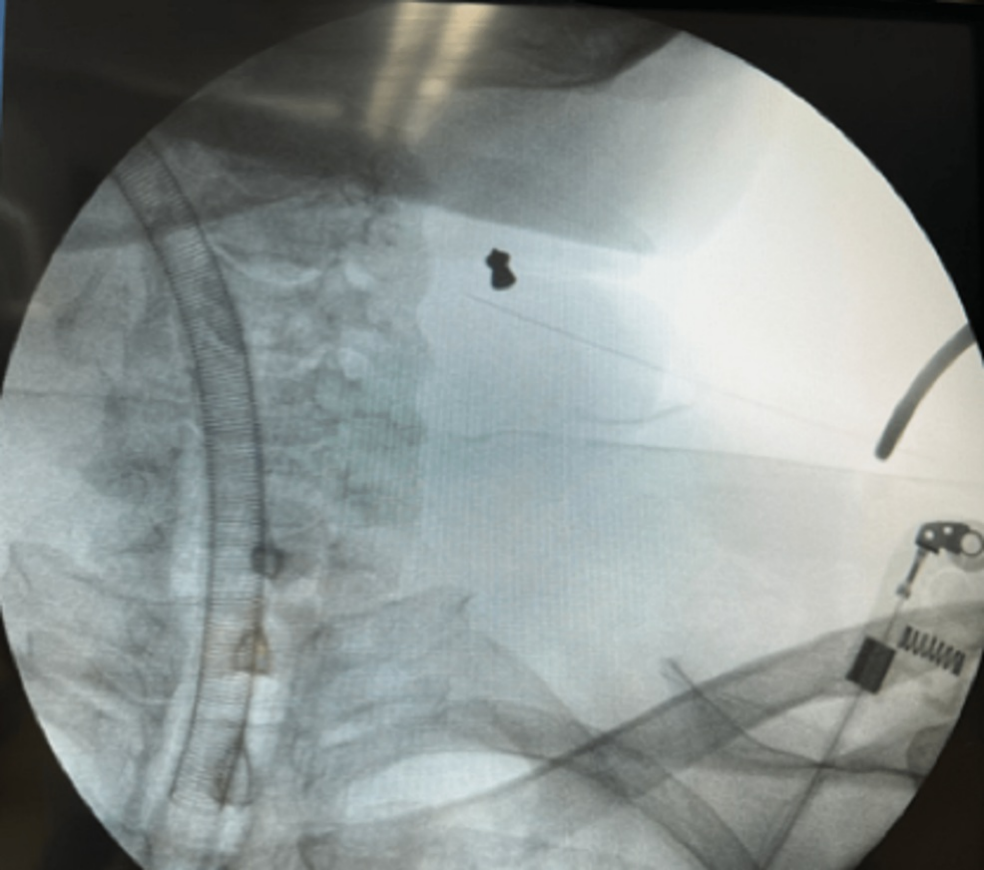
Under fluoroscopy guidance, the spinal needle was used for direct access localization of the metallic foreign body

**Figure 6:**
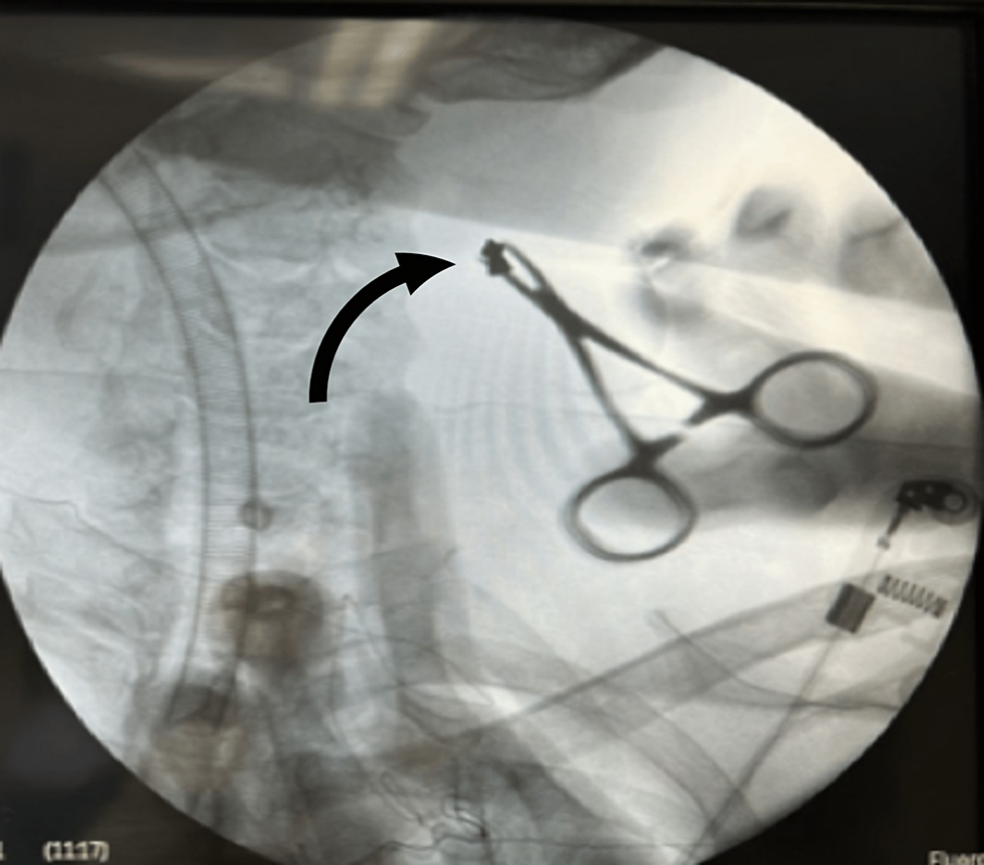
Fluoroscopy-guided removal of metallic foreign body

CT scan showed a foreign body within the sternocleidomastoid muscle on the right side. He was taken to the operating room and localization of the MFB was done using C-arm fluoroscopy (Figure 5) with a spinal needle for proper localization and direct access. A 0.5 cm skin incision was done and, using forceps under the C-Arm fluoroscopy, the MFB was removed (Figure 6). There were no complications, and the patient was discharged home on the same day.

## Discussion

Metabolic forging, a process that involves the manipulation of biological systems to enhance physical performance, has gained significant attention in recent years. One area of concern is the potential need for the removal of foreign bodies introduced during metabolic forging procedures. In this discussion, we will explore the use of fluoroscopy as a means of guiding the removal of such foreign bodies, with references to the relevant literature.

Fluoroscopy is an imaging technique that employs X-rays to produce real-time images of the internal structures of the body. It has been widely used in various medical procedures, such as angiography, orthopedic surgery, and gastrointestinal studies [11]. The use of fluoroscopy in the context of metabolic forging body removal is relatively new and has been gaining traction as a viable option for safe and effective removal.

Diagnosis

Accurately locating an MFB is essential for making a clinical decision. The location, type, and dimensions of the MFB may be revealed via a thorough historical and physical investigation. If the entrance was recently punctured, a visual inspection will reveal the wound. Other direct locating techniques such as CT, USG, and X-rays can identify the MFB and reveal further information about the relationship between it and the tissues or organs around it. Intraoperative C-arm fluoroscopy or USG may offer real-time images to locate the migrated foreign body. Several previous studies have reported the viability of C-arm fluoroscopy as a tool for detecting multiple metallic objects like broken dental needles. In addition, multiple studies were conducted showing the ability of X-ray fluoroscopy to detect small MFBs in the esophagus. The precise position of the foreign body in the surgical area is found using radiologic instruments. C-arm fluoroscopy can offer three-dimensional, real-time intraoperative images for the precise positioning of target materials, particularly when a foreign body is present in soft tissue and can be moved throughout the procedure by traction. Metal, gravel, and glass can all be reliably found using mini C-arm fluoroscopy, but wood or plastic cannot.

In a foreign body occurrence, the standard radiologic and CT images are routinely examined, and they can provide some insight into the whereabouts of the missing item. Additionally, visual endoscopy has been developed to find and get rid of foreign objects in the head and neck area. Even with the best imaging modalities, preoperative localization of a foreign body is usually challenging. The best inquiry to confirm the presence of a foreign body and provide a location for it is a CT of the neck in 1-mm cuts. Even on CT, it is occasionally impossible to tell whether a foreign body is completely extraluminal or only partially so. Additionally, one should be aware that the head and neck's position during surgery may change. Since the soft tissues of the neck are movable in comparison to the bony and cartilaginous structures, a foreign body found during surgery may not be in the same location as shown on CT [1]. 

Soft-tissue embedded metals can be defined and removed using C-arm fluoroscopy, which can provide three-dimensional intraoperative real-time images for the precise location of target materials, particularly when a foreign body in soft tissue is movable by intraoperative traction. Patients and operators are exposed to radiation within commonly used radiation limits when using C-arm fluoroscopy to define foreign bodies in the oral and maxillofacial area [4]. There are numerous ways to pinpoint an object inside soft tissue. There are several imaging techniques for locating foreign bodies, including plain radiographs, xeroradiographs, CT, MRI, electromagnetic metal detectors, and USG. These include grid systems, sinography, sonography, fluoroscopy, and stereotactic guides [12].

Before any surgical attempt, a CT scan is required to assess the status of the foreign body and nearby structures. An external approach is preferred for foreign bodies with complications or that are close to great vessels and vital organs [13]. Surgical magnets are low-cost and can localize objects that are non-palpable and lack characteristic USG features such as shadowing or the ring sign. They also avoid the risk of ionizing radiation associated with fluoroscopy [14]. 

Management

The anatomic site of pellet entry determines the type and severity of the injury. When a pellet enters the cranium, it can cause intracranial bleeding, CSF leakage, meningitis, brain abscess, traumatic aneurysm formation, and carotid-cavernous sinus fistula. Pellets can become lodged in the jawbones or paranasal sinuses as a result of facial injuries. When the pellets are easily accessible surgically, such as in the maxillary sinus, they can be easily removed using either conventional or endoscopic surgery using the Caldwell Luc approach, with minimal complications and hospital stay. However, when the pellets become embedded in deeper and more vital structures, the surgical procedure can be extremely invasive, resulting in significant morbidity and a lengthy hospital stay, possibly necessitating intensive care unit care [2]. 

Severe neck injuries have a high fatality rate. The treatment for these wounds depends on whether the patient exhibits "hard signs" such as airway obstruction, an air bubble wound, an expanding or pulsatile hematoma, active bleeding, hypovolemic shock, hematemesis, and neural deficits or hemodynamic instability during the initial examination [15]. 

An external neck approach has been used to remove foreign bodies in the para-pharyngeal space in the majority of cases because it allows visualization and completes surgical control of the carotid artery. The intraoral approach, on the other hand, has been reported as safe and effective, with no increased risk of vascular injuries, owing in large part to current medical imaging capabilities. This approach, in particular, can reduce the surgical burden and should be advocated for lesions located medial to the great vessels or close to the skull base [16]. 

There are many management guidelines for penetrating neck trauma; typically, the presence of hard signs necessitates exploratory surgery. However, retained foreign bodies in asymptomatic patients are a controversial entity, and their management necessitates careful consideration and judgment [15]. 

Fluoroscopy is a widely accessible, minimally invasive, but underutilized procedure. It offers an accurate intraoperative assessment of the foreign body in real-time. To allow the planning of a secure extraction pathway, the target should be radiopaque, and the surgeon should be knowledgeable about the anatomy of the neck. The fluoroscopic method is particularly useful for horizontally oriented foreign bodies and those with substantial extraluminal portions, both of which could be exceedingly difficult using an endoscopic approach when massive mucosal damage is hardly preventable. When the intraluminal portion of a foreign body inserted vertically is long enough to be seen during an endoscopy, removal can be accomplished via flexible pharyngo-laryngoscopy or upper gastrointestinal endoscopy. Rigid pharyngo-laryngoscopy or esophagoscopy are preferred for those who could not tolerate removal under local anesthesia [13].

Advantages of fluoroscopy

This real-time guidance reduces the risk of damaging nearby structures and ensures that the entirety of the foreign body is removed, preventing potential complications. Furthermore, fluoroscopy is a minimally invasive technique, which translates to reduced trauma to the patient, shorter recovery times, and a lower risk of infection compared to more invasive procedures [17]. The ability to perform the removal under local anesthesia also allows for better patient comfort and compliance. Details of reported cases of MFB that were removed under the fluoroscopy guidance are given in Table 1.

**Table 1:** Reported cases of fluoroscopy-guided metallic foreign body removal

Name of study	Publication year	Number of cases	Name of first author	Operation	Type of foreign body
1- Use of fluoroscopic guidance to remove a migrating esophageal foreign body	2015	1 case	Sinha et al. [1]	intraoperative fluoroscopic guidance, A C-arm fluoroscope was directed at the neck.	Metal wire
2- Successful localization of intraoral foreign body with c-arm fluoroscopy	2014	1 case	King et al. [4]	Location of the foreign body was determined by using C-arm fluoroscopy, and then was removed.	Metal screw in the intrinsic tongue muscle
3- Successful localization and surgical removal of ingested sewing needles under mini c-arm fluoroscopy: a case report	2006	1 case	Ma et al. [6]	Needle at the larynx was removed by a laryngoscope. Mini C-arm fluoroscopy was used to localize the remaining needles and removed all intraoperatively.	Sewing needles
4- Image-guided percutaneous removal of ballistic foreign bodies secondary to air gun injuries	2018	1000 cases	Rothermund et al. [9]	Image-guided percutaneous removal of ballistic foreign bodies. The primary modality to assist in removal was USG, closely followed by fluoroscopy.	Ballistic foreign bodies secondary to air gun injuries
5- Clinical approaches to migrating ingested foreign bodies in the neck	2022	2 cases	Ho et al. [13]	Horizontally-oriented pharyngeal fish bone with a portion in the neck, which was removed under fluoroscopic guidance and rigid laryngopharyngoscopy in succession.	Fishbone

Challenges and limitations

Despite its advantages, there are challenges and limitations associated with using fluoroscopy for metabolic forging body removal. One such limitation is the radiation exposure associated with fluoroscopy. Although the doses are generally low, prolonged or repeated exposure can pose risks to both the patient and the medical staff [18]. Therefore, it is essential to adhere to the "as low as reasonably achievable" (ALARA) principle when using fluoroscopy [19]. 

## Conclusions

Fluoroscopy-guided removal of foreign bodies related to metabolic forging is a promising technique with several advantages, including real-time visualization, reduced invasiveness, and shorter recovery times. However, it is essential to weigh the benefits against the risks associated with radiation exposure and inherent limitations in detecting non-metallic objects. Further research and clinical studies are needed to optimize this technique and establish evidence-based guidelines for its application in the field of metabolic forging bodies.
